# Prehemorrhage antiplatelet use in aneurysmal subarachnoid hemorrhage and impact on clinical outcome

**DOI:** 10.1177/17474930211035647

**Published:** 2021-07-29

**Authors:** Martina Sebök, Isabel C Hostettler, Emanuela Keller, Ilari M Rautalin, Bert A Coert, William P Vandertop, René Post, Ali Sardeha, Maud A Tjerkstra, Luca Regli, Dagmar Verbaan, Menno R Germans

**Affiliations:** 1 Department of Neurosurgery, University Hospital Zurich, Zurich, Switzerland; 2 Clinical Neuroscience Center, University Hospital Zurich, University of Zurich, Zurich, Switzerland; 3 Stroke Research Centre, University College London, Institute of Neurology, London, UK; 4 Department of Neurosurgery, Klinikum rechts der Isar, Munich, Germany; 5 Neurosurgical Intensive Care Unit, Department of Neurosurgery and Institute of Intensive Care Medicine, University Hospital Zurich, Zurich, Switzerland; 6 Department of Neurosurgery, Helsinki University Hospital, Helsinki, Finland; 7 Department of Neurosurgery, Amsterdam University Medical Center, Amsterdam, the Netherlands

**Keywords:** Aneurysmal subarachnoid hemorrhage, acetylsalicylic acid, antiplatelet agent, thrombocyte transfusion, outcome, rebleed, mortality

## Abstract

**Background:**

Literature is inconclusive regarding the association between antiplatelet agents use and outcome after aneurysmal subarachnoid hemorrhage.

**Aims:**

To investigate the association between clinical outcome and prehemorrhage use in aneurysmal subarachnoid hemorrhage patients as well as the impact of thrombocyte transfusion on rebleed and clinical outcome.

**Methods:**

Data were collected from prospective databases of two European tertiary reference centers for aneurysmal subarachnoid hemorrhage patients. Patients were divided into “antiplatelet-user” and “non-user” according to the use of acetylsalicylic acid prior to the hemorrhage. Primary outcome was poor clinical outcome at six months (Glasgow Outcome Scale score 1–3). Secondary outcomes were in-hospital mortality and impact of thrombocyte transfusion.

**Results:**

Of the 1033 patients, 161 (15.6%) were antiplatelet users. The antiplatelet users were older with higher incidence of cardiovascular risk factors. Antiplatelet use was associated with poor outcome and in-hospital mortality. After correction for age, sex, World Federation of Neurosurgical Societies score, infarction and heart disorder, pre-hemorrhage acetylsalicylic acid use was only associated with poor clinical outcome at six months (adjusted OR 1.80, 95% CI 1.08–3.02). Thrombocyte transfusion was not associated with a reduction in rebleed or poor clinical outcome.

**Conclusion:**

In this multicenter study, the prehemorrhage acetylsalicylic acid use in aneurysmal subarachnoid hemorrhage patients was independently associated with poor clinical outcome at six months. Thrombocyte transfusion was not associated with the rebleed rate or poor clinical outcome at six months.

## Introduction

Aneurysmal subarachnoid hemorrhage (aSAH) is a potentially fatal disease, carrying a six months’ case fatality rate of 55–60%^1–3^ and more than one third of survivors have severe disability.^4^ Many complications such as rebleed, delayed cerebral ischemia (DCI) and hydrocephalus are multifactorial and negatively affect clinical outcome.^3^

Antiplatelet agents are used for secondary prevention of various cardiovascular and cerebrovascular events in a wide range of high-risk patients,^5–7^ and their use has been associated with a lower incidence of aSAH. It is hypothesized that this might be mediated by a protective effect against chronic inflammation and subsequent aneurysm wall degeneration.^8–10^ However, antiplatelet use has also been related to early rebleeds, treatment-related complications and worse outcome after aSAH.^11–13^

Decision-making regarding the management of patients with prehemorrhage antiplatelet agents use is mainly based on inconsistent results and nonsignificant findings.^11–15^ As no evidence-based recommendations regarding the management of prehemorrhage antiplatelet use in aSAH patients exist, the decision to stop antiplatelet medication is often associated with the presence or absence of local guidelines.^16^ One recent study^17^ found an association between thrombocyte transfusion and poor clinical outcome after six months in patients with aSAH and this needs further clarification.

## Aims

The main purpose of this study was to investigate the influence of prehemorrhage antiplatelet use on the clinical outcome after aSAH, considering confounding factors. Additionally, we studied potential effects of thrombocyte transfusion on the clinical outcome and rebleed after aSAH.

## Methods

### Data collection

Patients were retrieved from prospectively collected databases, including patients at the Department of Neurosurgery of the University Hospital Zurich treated between January 2005 and December 2016 and the Academic University Medical Center Amsterdam treated between December 2011 and December 2015. Both hospitals are high-volume tertiary reference centers for the treatment of aSAH. The research ethics board of the Canton Zurich, Switzerland approved this study.

### Patient characteristics

We included patients older than 18 years of age who had confirmed aSAH on admission computed tomography (CT) imaging or positive lumbar puncture and a proven aneurysm in either computed tomography angiography or digital subtraction angiography. Subjects meeting any of the below-mentioned criteria were excluded from this study: patients with non-aneurysmal SAH, perimesencephalic hemorrhage (according to the previous published definition^18^) as well as patients with traumatic SAH.

Patients were divided into the groups “antiplatelet-user” and “non-user” according to the use of acetylsalicylic acid (ASA) prior to the hemorrhage. Because ASA in combination with other antiplatelet agents was assumed to be associated with a worse outcome than ASA alone, the outcome of this patient group was explored prior to including them to the “antiplatelet-user” cohort and excluded if they showed a significant difference in outcome. Patients with anticoagulation therapy were excluded on beforehand. ASA was stopped in all patients immediately after the radiological diagnosis of aSAH.

The patients’ characteristics, clinical and radiological data as well as clinical outcome data were collected by trained staff and verified by an attending vascular neurosurgeon. Furthermore, we collected the treatment modalities, in-hospital complications as well as cardiovascular risk factors (smoking, hypertension, hypercholesterolemia, heart disorder, diabetes). Heart disorders were characterized according to World Heart Federation.^19^ The initial clinical severity and radiological grade were assessed using the World Federation of Neurosurgical Societies (WFNS) grade and the Fisher score, respectively. We dichotomized the WFNS into WFNS 1–3 and 4–5 and the Fisher score into 3 vs. 1, 2 and 4.^20,21^

In-hospital complications including hydrocephalus and its treatment modality (external ventricular drainage (EVD) or ventriculoperitoneal (VP) shunt placement), rebleed, occurrence of DCI and infarction were registered for outcome comparison between groups. Only patients with radiologically confirmed rebleed were included in the rebleed group. DCI was defined according to Vergouwen et al.^22^ Only confirmed new ischemic lesions on follow-up imaging (CT and/or magnetic resonance imaging), which were not seen immediately after the aneurysm excluding procedure, were included in the analysis. The clinical outcome was evaluated using the Glasgow outcome scale (GOS), with GOS 1 (death) at initial hospital admission and six-months’ follow-up, and poor (GOS 1–3) and favorable (GOS 4–5) outcome at six-months’ follow-up.

### Data analysis

Prior to data analysis, we explored the outcome of patients with ASA in combination with other antiplatelet agents in relation patients with ASA alone. As all patients with double antiplatelet medications had a poor outcome, which was significantly different from the group with ASA alone, we decided to exclude these patients from further analysis.

Baseline characteristics, disease-associated complications, treatment and outcome factors were compared between “antiplatelet users” and “non-users.” Continuous variables are presented as mean with its standard deviation (SD) if normally distributed and as median with its interquartile range if not. Categorical variables are presented as count and percentages and are dichotomized to relevant clinical cut-off points. Group differences are calculated using the Chi-square test and Student’s t-test. A two-sided p-value < 0.05 is considered significant.

Crude and adjusted odd ratios (OR) were calculated for prehemorrhage antiplatelet use in relation to poor outcome, in-hospital mortality and mortality at six months with logistic regression analysis. If the change between crude and adjusted OR was >10%, the corresponding parameter for which the stratification was performed was considered as a confounder. A multivariable logistic regression analysis was performed, adjusting for confounders. In the antiplatelet user group, the impact of thrombocyte transfusion on rebleed and poor outcome was calculated using a Chi-square test. Analysis was performed with STATA version 16.0 (StataCorp, Stata Statistical Software: Release 16, College Station, TX) software.

## Results

### Baseline characteristics and treatment modalities

**Figure 1.**
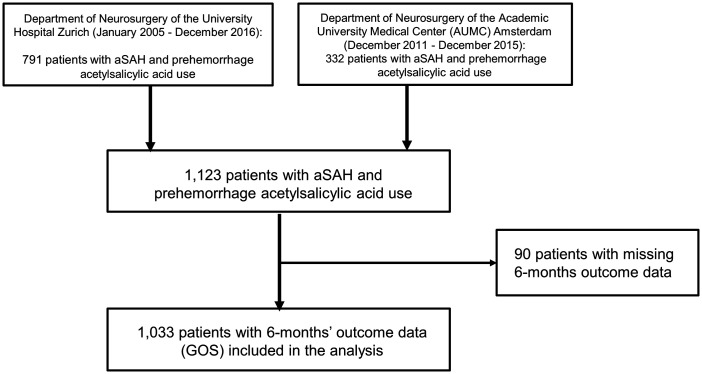
Study flow chart. Between January 2005 and December 2016, 791 patients with aSAH and prehemorrhage antiplatelet use were treated at the Department of Neurosurgery of the University Hospital Zurich and 332 patients between December 2011 and December 2015 at the Department of Neurosurgery of the Academic University Medical Center Amsterdam. Cumulative, 1123 patients with aSAH and prehemorrhage acetylsalicylic acid use were available for inclusion in this prospective cohort study. Ninety patients with missing six-month clinical outcome data were excluded from the study. In the final analysis, 1033 patients were included. aSAH: aneurysmal subarachnoid hemorrhage.

A total of 1123 patients were eligible for the study. Ninety patients (8.0%) with missing outcome data at six months were excluded from the analysis. The study flowchart is given in Figure 1. The remaining 1033 patients consisted of 692 (67%) women and 161 (15.6%) used ASA prior to the onset of aSAH (Table 1). Antiplatelet users were older compared to non-users with a higher prevalence of hypertension, diabetes, heart disorder and hypercholesterolemia (Table 1). No difference was seen in clinical status on admission, nor in the Fisher score between both groups (Table 1).

**Table 1.** Baseline characteristics of 1033 patients with aneurysmal subarachnoid hemorrhage.

	Antiplatelet users (n = 161)	Nonusers (n = 872)	p-value
Age (mean ± SD)	63.6 ± 12.5	54.4 ± 12.3	**<0.001**
Sex, female, n (%)	109 (67.7)	583 (66.9)	0.83
Smoking, n (%)	75 (46.6)	416 (50.7)	0.36
Hypertension, n (%)	94 (58.4)	263 (30.2)	**<0.001**
Hypercholesterolemia, n (%)	42 (26.1)	63 (7.2)	**<0.001**
Heart disorder, n (%)	55 (34.2)	53 (6.1)	**<0.001**
Diabetes, n (%)	14 (8.7)	28 (3.2)	**0.001**
Fisher score			0.74
= 1, n (%)	6 (3.7)	32 (3.7)	
= 2, n (%)	4 (2.5)	43 (4.9)	
= 3, n (%)	30 (18.6)	229 (26.3)	
= 4, n (%)	121 (75.2)	568 (65.1)	
WFNS			0.43
= 1, n (%)	57 (33.7)	287 (32.9)	
= 2, n (%)	35 (21.5)	222 (25.5)	
= 3, n (%)	13 (8.1)	98 (11.2)	
= 4, n (%)	29 (18.0)	136 (15.6)	
= 5, n (%)	27 (16.8)	129 (14.8)	
Aneurysm clipping, n (%)	67 (41.6)	357 (40.9)	0.87
Aneurysm coiling, n (%)	72 (44.7)	448 (51.4)	0.12
No treatment, n (%)	22 (13.7)	67 (7.7)	0.14

p-values in bold indicate significant difference. n: number; SD: standard deviation; WFNS: World Federation of Neurological Surgeons.

### Outcome

The proportion of poor outcome at six months’ follow-up was higher in the antiplatelet group compared to the non-user group (OR 2.45, 95% CI 1.74–3.47) (Supplementary Table 1). A significant increase of in-hospital mortality as well as increase in mortality at six months was observed in the antiplatelet user group (OR 1.94, 95% CI 1.34–2.82 and OR 1.87, 95% CI 1.28–2.73, respectively) (Supplementary Tables 2 and 3). We did not find a difference in rebleed rate, DCI, infarction, rate of hydrocephalus, need for urgent EVD or VP-shunt dependency (Table 2). In the antiplatelet user group, five patients were diagnosed with NSTEMI, most likely due to the aSAH itself.

**Table 2.** Clinical outcome, mortality and in-hospital complications and their treatment modalities of 1033 patients with aneurysmal subarachnoid hemorrhage.

	Antiplatelet users (n =161)	Nonusers (n = 872)	p-value
Poor outcome (GOS 1–3), n (%)	90 (55.9)	297 (34.1)	**<0.001**
In-hospital mortality, n (%)	49 (30.4)	165 (18.9)	**0.001**
Mortality at six months, n (%)	50 (31.1)	181 (20.8)	**0.004**
Rebleed, n (%)	22 (13.7)	102 (11.7)	0.49
Delayed cerebral ischemia, n (%)	32 (19.9)	219 (25.1)	0.16
Infarction (not post-treatment)^a^, n (%)	26 (22.4)	129 (21.0)	0.29
Hydrocephalus, n (%)	110 (68.3)	539 (61.8)	0.12
Urgent external ventricular drain, n (%)	91 (56.5)	453 (51.9)	0.29
Ventriculoperitoneal-shunt dependency, n (%)	41 (25.5)	178 (20.4)	0.15

p-values in bold indicate significant difference. All p-values were calculated using a Chi-square test. GOS: Glasgow Outcome Scale; n: number.

a For n=735 (antiplatelet users: 122; nonusers: 613).

### Risk factors and poor outcome at six months

The following parameters were assessed as confounders and are included into the multivariable analysis (Supplementary file 1, 2 and 3): age, sex, WFNS score, infarction and heart disorder for poor outcome and age, sex, infarction, heart disorder, hypercholesterolemia and smoking for in-hospital and six months’ mortality. Antiplatelet use was independently associated with poor outcome at six months (adjusted OR 1.80, 95% CI 1.08–3.02), whereas it was neither associated with in-hospital mortality (adjusted OR 0.97, 95% CI 0.56–1.66) nor with mortality at six months (adjusted OR 0.89, 95% CI 0.51–1.53).

### Thrombocyte transfusion

Of the 161 patients with a history of prehemorrhage ASA use, 67 (41.6%) received a thrombocyte transfusion. Patients who received a thrombocyte transfusion after the rebleeding event (n = 2) were not included in the transfusion group. The rate of rebleed was higher in patients who did not receive a thrombocyte transfusion; however, this difference was not significant (transfusion vs. no transfusion: 5 (7.7%) vs. 17 (17.7%), p = 0.07; Table 3). There was no difference in poor outcome between patients receiving thrombocyte transfusion and those who did not (transfusion vs. no transfusion: 37 (55.2%) vs. 53 (56.4%), p = 0.83; Table 3).

**Table 3.** Crosstab of thrombocyte transfusion impact (in patients with pre-hemorrhage antiplatelet use) on rebleed rate and poor outcome (GOS 1–3).

	Thrombocyte transfusion		
	Yes	No	p-value
Rebleed rate, n (%)	5/65^a^ (7.7%)	17/96 (17.7%)	0.07
Poor outcome, n (%)	37/67 (55.2%)	53/94 (56.4%)	0.83

GOS: Glasgow Outcome Scale; n: number.

a Given the fact that we wanted to address aneurysm rebleeding cases, the two patients, who received a thrombocyte transfusion after the rebleeding event were for this crosstab calculation included in the non-transfusion group.

## Discussion

In this multicenter study, the prehemorrhage ASA use in aSAH patients was identified as an independent risk factor for poor outcome (defined as GOS 1–3) at six months after aSAH. Thrombocyte transfusion in the subgroup of antiplatelet users had no significant impact on the rate of rebleed or patient outcome at six months.

### Impact of prehemorrhage ASA use on clinical outcome after aSAH

Previous studies have reported conflicting results regarding the impact of prehemorrhage antiplatelet use and patients’ outcome and some did not find an association between ASA use (other antiplatelet or anticoagulant agents were excluded) and poor outcome.^12^ Our data show a significantly higher proportion of poor outcome and in-hospital mortality in the ASA users. This cannot be explained by a higher rebleed rate, nor by a more severe hemorrhage pattern. Our finding that prehemorrhage antiplatelet use is an independent risk factor for poor outcome has previously been suggested.^11^ Kato et al. reported the influence of antiplatelet agents before the onset of hemorrhage and concluded that antiplatelet agents was significantly associated with worse outcome in patients of 70–79 years.^11^

### Cerebrovascular and cardiovascular effects of antiplatelet agents

Antiplatelet agents, especially ASA, have well-known positive effects on reducing the risk of cardiovascular^23,24^ and cerebrovascular^25,26^ diseases. Moreover, several recent studies suggested that ASA may decrease the risk of growth and rupture of cerebral aneurysms.^8,10,27^ After rupture, however, ASA is possibly associated with an increased risk of recurrent bleeding before treatment^28^ and ASA given after aneurysm treatment in aSAH does not improve clinical outcome.^29^

One study showed that long-term ASA and anticoagulant use among patients with aSAH and endovascular aneurysm treatment was not associated with increased mortality or complication rates.^14^ Another study suggested a potential beneficial effect of ASA in the setting of intracranial aneurysms by weighing the risk of rupture against its potential adverse effects on hemorrhage severity. Those findings contradict with our findings, however, both studies drew their conclusions on a lower number of patients.

A possible reason for the increased risk of poor outcome in antiplatelet users could be the inhibition of platelet activation. This can theoretically exacerbate the initial hemorrhage and the use of antiplatelet drugs can complicate surgical procedures.^11^ However, in our cohort we could not confirm this hypothesis as we did not find a significant difference in rebleed rate. Furthermore, higher age and higher proportion of heart disorder in antiplatelet users could have influenced the outcome. However, when we corrected for these confounders, antiplatelet use remained associated with poor outcome. This is opposing to findings by Bruder et al.^30^ who in the matched-pair analysis did not find a different outcome as evaluated by modified Rankin scale between patients with continuous ASA and patients without ASA.

Due to the aging population and rising number of patients with cardiovascular or neurovascular disease, there is an increasing number of patients taking antiplatelet and anticoagulative drugs.^31^ A previous study showed a significant increase in the rate of patients with continuous ASA use at the time of aneurysm rupture over the observed period of 15 years.^30^ This emphasizes the importance of guidelines and treatment recommendations in this patient group. A recently conducted survey shows that there is significant variability in the management of patients with aSAH and antiplatelet use before admission.^16^ Departmental guidelines are only present in 32% and have an impact on decision-making to stop the antiplatelet agent and/or transfuse thrombocytes.^16^

### Impact of thrombocyte transfusion on outcome after aSAH

Thrombocyte transfusion in patients with spontaneous intracerebral hemorrhage has been investigated in several studies but patients with aSAH were excluded.^27,32,33^ Results of the randomized controlled PATCH trial^34^ showed a higher case fatality rate when thrombocytes were acutely transfused in non-surgically treated patients taking antiplatelet therapy prior to intracerebral hemorrhage. Although patients with aSAH were not included in the study, it opened the discussion about whether thrombocyte transfusion in aSAH could be harmful to patients instead of beneficial. In a recent consecutive series of 364 patients with aSAH, 38 patients used antiplatelet therapy prior to admission and underwent thrombocyte transfusion during hospital admission; those patients showed poor clinical outcome at six months after correcting for confounders.^17^ Based on the available data, however, no firm recommendation regarding thrombocyte transfusion in patients with aSAH can be given so future research in larger cohorts are needed.

No difference in poor outcome at six months between the patients with and without transfusion was seen. So, based on the findings of our study, thrombocyte transfusion does not seem to be harmful in aSAH patients. On the contrary, a recent study by Post et al. found an association between thrombocyte transfusion and poor clinical outcome at six months.^17^ Based on current very limited findings, we still do not know the balance between risk and benefit of thrombocyte transfusion in patients with aSAH and prehemorrhage antiplatelet use. A pooled analysis of data from both cohort and larger international (randomized) studies could clarify this issue.

### Limitations

We performed a retrospective analysis of prospectively collected data and some data were collected retrospectively. This way of data collection could have induced information bias. Moreover, information bias could also have been introduced by failure in recalling whether the patient used ASA by the next of kin. To minimize the information bias, the validated outcome measurements were collected by physicians and research nurses who were all trained for performing outcome assessment. Since studies have found that 25% of all patients with aSAH die before reaching hospital, our data are not generalizable to the complete aSAH population including non-hospitalized patients.^35^ We did not collect data on duration of ASA use before the hemorrhage, neither did we assess thrombocyte aggregation status. Finally, the timing of thrombocyte transfusion was not assessed in the current cohort.

## Conclusion

In this multicenter study, the use of ASA before onset of aSAH was found to be independently associated with poor outcome (defined as GOS 1–3) at six months. Prehemorrhage ASA use is not associated with a higher in-hospital or six months’ mortality rate. Thrombocyte transfusion had no impact on the rebleed rate or poor outcome at six months. Future studies are necessary to assess the optimal management of patients who use ASA with or without other antiplatelet agents before the onset of aSAH.

## Supplemental Material

sj-pdf-1-wso-10.1177_17474930211035647 - Supplemental material for Prehemorrhage antiplatelet use in aneurysmal subarachnoid hemorrhage and impact on clinical outcome Click here for additional data file. Supplemental material, sj-pdf-1-wso-10.1177_17474930211035647 for Prehemorrhage antiplatelet use in aneurysmal subarachnoid hemorrhage and impact on clinical outcome by Martina Sebök, Isabel C Hostettler, Emanuela Keller, Ilari M Rautalin, Bert A Coert, William P Vandertop, René Post, Ali Sardeha, Maud A Tjerkstra, Luca Regli, Dagmar Verbaan and Menno R Germans in International Journal of Stroke
