# Supplementary material for: Prehemorrhage antiplatelet use in aneurysmal subarachnoid hemorrhage and impact on clinical outcome
Source: Int J Stroke. 2021 Jul 29;17(5):545–52. doi: 10.1177/17474930211035647 (PMC9150139; doi:10.1177/17474930211035647)
Supplement: sj-pdf-1-wso-10.1177_17474930211035647 - Supplemental material for Prehemorrhage antiplatelet use in aneurysmal subarachnoid hemorrhage and impact on clinical outcome [file sj-pdf-1-wso-10.1177_17474930211035647.pdf]

Supplementary Material

Supplementary Table 1: Logistic regression analysis for poor outcome defined as GOS 1-3 for whole cohort and controlled for age sex, WFNS≤ 3, infarction and heart disorder

**Risk**	**Univariate analysis**			**Multivariate analysis**		
	OR	*95% CI*	*p-value*	*aOR*	*95% CI*	*p-value*
**Antiplatelet**	2.45	1.74-3.47	<0.001	**1.80**	**1.08-3.02**	**0.03**
**Center**	1.20	0.91-1.58	0.20			
**Age (linear)**	1.04	1.03-1.05	<0.001	1.04	1.02-1.05	<0.001
**Sex**	0.93	0.71-1.23	0.61			
**Posterior circulation aneurysm**	1.50	1.07-2.10	0.02			
**Fisher score = 3**	0.34	0.24-0.48	<0.001			
**WFNS ≤ 3**	0.12	0.09-0.17	<0.001	0.12	0.08-0.17	<0.001
**Hydrocephalus**	3.73	2.74-5.09	<0.001			
**DCI**	2.04	1.53-2.74	<0.001			
**Rebleed**	4.40	2.91-6.67	<0.001			
**Infarction**	4.12	2.79-6.07	<0.001	4.23	2,74-6.51	<0.001
**Heart disorder**	2.02	1.35-3.02	<0.001	1.07	0.55-2.09	0.84
**Hypertension**	1.16	0.89-1.51	0.26			
**Diabetes**	1.26	0.68-2.36	0.46			
**Hypercholesterinemia**	0.90	0.59-1.37	0.62			
**Smoking**	0.62	0.48-0.82	0.001			
**Clipping**	1.02	0.79-1.32	0.88			
**Coiling**	0.39	0.30-0.51	<0.001			

aOR: adjusted odds ratio, CI: confidence interval, DCI: delayed cerebral ischemia, GOS: Glasgow Outcome Scale, OR: odds ratio, WFNS: World Federation of Neurological Surgeons

Supplementary Table 2: Logistic regression analysis for in-hospital mortality for whole cohort and controlled for age, sex, infarction, heart disorder, hypercholesterinemia and smoking.

**Risk**	**Univariate analysis**			**Multivariate analysis**		
	OR	*95% Cl*	*p-value*	*aOR*	*95% Cl*	*p-value*
**Antiplatelet**	1.94	1.34-2.82	<0.001	**0.97**	**0.56-1.66**	**0.91**
**Center**	1.41	1.04-1.92	0.03			
**Age (linear)**	1.04	1.03-1.05	<0.001	1.03	1.01-1.05	<0.001
**Sex**	1.18	0.87-1.60	0.28	1.23	0.82-1.85	0.33
**Posterior circulation aneurysm**	1.65	1.15-2.39	0.001			
**Fisher score = 3**	0.40	0.26-0.61	<0.001			
**WFNS ≤ 3**	0.16	0.11-0.23	<0.001			
**Hydrocephalus**	2.78	1.96-3,94	<0.001			
**DCI**	1.69	1.23-2.32	0.001			
**Rebleed**	3.59	2.42-5.33	<0.001			
**Infarction**	2.83	1.9-4.21	<0.001	2.94	1.96-4.40	<0.001
**Heart disorder**	2.14	1.39-3.28	<0.001	1.89	1.02-3.47	0.04
**Hypertension**	1.11	0.82-1.50	0.55			
**Diabetes**	1.19	0.60-2.37	0.62			
**Hypercholesterinemia**	0.69	0.40-1.18	0.18	0.59	0.28-1.26	0.17
**Smoking**	0.69	0.50-0.96	0.03	0.65	0.43-0.97	0.04
**Clipping**	0.49	0.36-0.68	<0.001			
**Coiling**	0.41	0.30-0.56	<0.001			

aOR: adjusted odds ratio, CI: confidence interval, DCI: delayed cerebral ischemia, OR: odds ratio, WFNS: World Federation of Neurological Surgeons

Supplemental table 3: Logistic regression analysis for mortality at six months for whole cohort and controlled for age, sex, infarction, heart disorder, hypercholesterinemia and smoking.

Risk	Univariate analysis			Multivariate analysis		
	OR	*95% Cl*	*p-value*	*aOR*	*95% Cl*	*p-value*
**Antiplatelet**	1.87	1.28-2.73	<0.001	**0.89**	**0.51-1.53**	**0.67**
**Center**	1.25	0.90-1.72	0.18			
**Age (linear)**	1.04	1.03-1.05	<0.001	1.03	1.02-1.05	<0.001
**Sex**	1.22	0.88-1.66	0.23	1.30	0.85-1.97	0.23
**Posterior circulation aneurysm**	1.64	1.13-2.40	0.01			
**Fisher score = 3**	0.34	0.22-0.54	<0.001			
**WFNS ≤ 3**	0.15	0.11-0.22	<0.001			
**Hydrocephalus**	2.84	1.96-4.10	<0.001			
**DCI**	1.66	1.19-2.31	0.003			
**Rebleed**	3.10	2.07-4.65	<0.001			
**Infarction**	2.97	1.98-4.48	<0.001	3.14	2.07-4.76	<0.001
**Heart disorder**	2.10	1.36-2.24	<0.001	1.96	1.05-3.63	0.03
**Hypertension**	1.08	0.79-1.48	0.62			
**Diabetes**	1.21	0.58-2.49	0.61			
**Hypercholesterinemia**	0.66	0.38-1.16	0.14	0.58	0.27-1.25	0.16
**Smoking**	0.68	0490-0.96	0.03	0.69	0.45-1.04	0.08
**Clipping**	0.50	0.36-0.70	<0.001			
**Coiling**	0.38	0.27-0.52	<0.001			

aOR: adjusted odds ratio, CI: confidence interval, DCI: delayed cerebral ischemia, OR: odds ratio, WFNS: World Federation of Neurological Surgeons
